# Quality Assessment of Complementary Feeding (CF) Practices Using CF Index and Determining Its Association With Sociodemographic Factors Among Children Aged 6-23 Months in District Etawah, Uttar Pradesh, India: A Community-Based Cross-Sectional Study

**DOI:** 10.7759/cureus.113808

**Published:** 2026-08-02

**Authors:** Balram Prajapati, Adarsh Maurya, Neelam Prajapati, Vidya Rani, Naresh P Singh, Pankaj Jain, Gagan Kaur, Sushil Shukla

**Affiliations:** 1 Community Medicine, Uttar Pradesh University of Medical Sciences (UPUMS), Saifai, IND; 2 Community Medicine, Uma Nath Singh Autonomous State Medical College, Jaunpur, IND; 3 Physical Medicine and Rehabilitation, King George's Medical University (KGMU), Lucknow, IND

**Keywords:** complementary feeding, complementary feeding index, dietary diversity, infant and young child feeding, malnutrition

## Abstract

Background: Achieving nutritionally sufficient and well-timed supplementary feeding is central to sustaining physical growth, brain maturation, and immunological competence in infants and toddlers from 6 through 23 months. The Complementary Feeding Index (CFI) functions as an integrated multi-component instrument designed to quantify the overall adequacy of young child nutrition at the household level.

Objective: This investigation sought to appraise complementary feeding adequacy via the CFI and explore its associations with sociodemographic characteristics among children between 6 and 23 months of age in Etawah district, Uttar Pradesh, India.

Methods: This community-based cross-sectional analytical study recruited 416 children aged 6-23 months across rural and urban localities of Etawah district between January 2024 and February 2026. Household data were obtained through a validated, pre-piloted structured interview tool. CFI sub-indicators were evaluated: breastfeeding continuation, bottle use, age-appropriateness of complementary food introduction, dietary variety, food group coverage, and meal frequency. Anthropometric indices were measured with calibrated instruments, and all data were processed in Statistical Product and Service Solutions (SPSS, version 26.0; IBM SPSS Statistics for Windows, Armonk, NY).

Results: Breastfeeding was ongoing in 70.9% of children; 35.6% were bottle-fed. Supplementary foods at the recommended six-month milestone were given in 52.2% of cases. Minimum dietary diversity (MDD) was met by 22.1%, minimum meal frequency (MMF) by 59.4%, and minimum acceptable diet (MAD) by only 13.9%. Overall, CFI classification placed 43% of children in the appropriate and 57% in the inappropriate feeding category.

Conclusion: Supplementary feeding quality in Etawah district is far below recommended thresholds, with 57% the children unable to achieve adequate dietary diversity or a MAD. Sustained investment in nutrition counselling, frontline workforce skill-building, and locally tailored community programmes is essential to reverse these deficits and improve child nutrition.

## Introduction

The 6-23 months age window is a critical period of nutrition as accelerated somatic growth, active neurological maturation, and emerging immune function make exclusively breastfeeding insufficient to meet the child's growing micronutrient and energy needs [1,2]. Therefore, introduction of safe, developmentally appropriate, and nutritious foods along with continued breastfeeding after about six months of age is essential to ensure healthy physical and cognitive growth and prevent long-term disease burden [3,4]. Undernutrition in childhood remains a major public health problem: currently, there are an estimated 148 million stunted children, 45 million wasted, and 37 million overweight children, and all these children take up almost half of all children below five years old, accounting for almost half of all mortality [5,6]. About 35.5% of children in India are stunted, 19.3% wasted, and 32.1% underweight, with less than one in 10 children aged 6-23 months meeting the minimum acceptable diet (MAD) and nearly one in four (24.1%) not reaching the minimum dietary diversity (MDD). Uttar Pradesh does worse with only 5.9% of young children reaching MAD thresholds [7]. Elevated undernutrition prevalence and feeding patterns of predominantly cereal-based staple foods with limited dietary diversity exist in Etawah district, as well as analogous conditions [8-10].

The Complementary Feeding Index (CFI) is a multi-component, integrative assessment where breastfeeding, food introduction timing, dietary variety, and meal adequacy are measured concurrently, allowing for more fine-grained identification of problems with food introduction practices [11,12]. Despite the ongoing efforts of the government through the Integrated Child Development Services (ICDS) and the POSHAN Abhiyaan National Nutrition Programme, the infant and young child feeding (IYCF) situation in Uttar Pradesh remains sub-optimal, and data are limited, especially at the district level, on the situation in Etawah. The CFI can be used to identify modifiable gaps and provide precision interventions, to build the capacity of frontline workers, and to help achieve Sustainable Development Goal 2 (SDG-2, Zero Hunger) and SDG-3 (Good Health and Well-Being).

## Materials and methods

This study used the cross-sectional (community-based) analytical design that included both rural and urban areas of Etawah district of Uttar Pradesh, India, with a total population of 1,581,810 (23.16% urban and 76.84% rural) as per the 2011 census [13]. Data collection was carried out continuously for 26 months from January 2024 to February 2026. The number of samples was determined by a reference prevalence of complementary feeding adequacy obtained from a comparable population in North India (51.3%), following the standard single proportion formula at 95% confidence and 5% precision, which resulted in 384 (rounded up to 416 to account for accuracy) [14]. There were 208 children from each rural and urban strata. A two-stage probability sampling design was employed in rural areas: in the first stage, two villages were drawn by lottery from each of the eight community development blocks (CDBs), resulting in 16 villages; in the second stage, 13 children of the eligible age were sequentially selected by house-to-house canvassing until each stratum (village) quota was reached. In the urban localities, multistage random selection was used - 16 wards in the municipality were selected (out of 41 wards), and within the selected wards, a residential colony was randomly selected. Thirteen eligible children per residential colony were then recruited through door-to-door visits.

Eligible children were those 6-23 months of age with a parent/legal guardian who provided written informed consent prior to enrolment. Children were excluded if they had any structural congenital defect (cleft lip/palate), a known diagnosis of cerebral palsy or chronic systemic disease, or were already receiving therapeutic feeding for severe acute malnutrition (SAM). Households with no main caregiver or that did not provide consent at the visit were also omitted.

Data collection tools and procedure

A structured questionnaire with five topical modules that was pre-tested and field pre-tested in the field by an interviewer was employed in collecting data. The sociodemographic characteristics of households were measured with the household sociodemographic variables in module one, such as a household socioeconomic status (HSS), caste, religion, and the family structure, and individual-level variables, such as age, sex, and the birth order of the child. The composite CFI that included six complementary feeding indicators (CFI) (continuation on breastfeeding, bottle use, timely complementary food introduction, food group frequency, frequency of meals, and dietary diversity) was used to measure complementary feeding quality in module two (Appendix). Each indicator was awarded one of two different points (1 = suboptimal; 2 = adequate), and his/her CFI total was found by combining the individual scores. Those children whose scores were below the sample mean CFI scores were considered to have inappropriate feeding, and those whose scores were at or above the sample mean CFI scores were considered to have appropriate feeding. This study involved expert content validation, piloting (10% of the expected sample size), daily checking by the principal investigator of completed questionnaires, and random supervisory re-verification (10% of records). The study obtained ethical clearance from the Institutional Ethics Committee of UPUMS. All caregivers were given the opportunity to voluntarily provide their written consent in the local language, and participants' anonymity and data confidentiality were maintained throughout. Data were analysed in Statistical Product and Service Solutions (SPSS, version 26.0; IBM SPSS Statistics for Windows, Armonk, NY) using descriptive statistics (frequencies, proportions, mean value, and standard deviation) and chi-square statistics to test for association of sociodemographic factors with the feeding classification at a 5% significance level with 95% confidence intervals.

## Results

Table 1 shows the sociodemographic characteristics of the total children and family members (416) enrolled. Most of the children (350, 84.13%) were in the age band 9-23 months, and a few (66, 15.87%) were in the age band six to eight months. Males outnumbered the females (228, 54.8% males; 188, 45.2% females). The most common birth order rank was second rank (160, 38.5%), followed by birth order ≥3 (144, 34.6%), and first rank (112, 26.9%). The majority of mothers (237, 57.0%) were between 21 and 30 years old. The educational status of the mothers was mainly in the range of junior to intermediate (combined illiteracy and basic literacy was 29, 7.0%), and homemaking was the single most common occupation (327, 78.7%). For fathers, 234 (56.3%) were over 30 years old; education was mostly secondary to intermediate, with a large number of fathers being graduates of this level; and the most common occupation was self-employed (225, 54.1%). There are 358 (86.1%) Hindu households with other backward classes (OBC, dominant caste group: 208, 50.0%), followed by general (138, 33.2%) and scheduled castes/scheduled tribes (SC/ST)/others (70, 16.8%). The majority of families were nuclear (228, 54.8%), followed by joint (154, 37.0%). It was observed that 172, 41.3% of the respondents were of socioeconomic class IV, and 164 (39.4%) belonged to socioeconomic class III, which indicated that the majority of the respondents were of lower middle-class economic status.

**Table 1:** Sociodemographic profile of the study participants *Birth order ≥3 includes third and higher-order births. ** Other religions include Christian, Jain, Buddhist, Parsi, etc. ***Modified BG Prasad Classification (January 2025), CPI-IW = 143.2

S. No.	Variable	Category	Frequency (n)	Percentage (%)
1.	Age of child (months)	6-8 months	66	15.87
		9-23 months	350	84.13
2.	Gender	Male	228	54.8
		Female	188	45.2
3.	Birth order	1	112	26.9
		2	160	38.5
		≥3*	144	34.6
4.	Age of the mother (years)	<20	49	11.8
		21-30	237	57.0
		>30	130	31.2
5.	Education of the mother	Illiterate	17	4.1
		Just literate	12	2.9
		Primary (5th)	32	7.7
		Junior high (8th)	88	21.2
		High school (10th)	93	22.4
		Intermediate (12th)	107	25.7
		Graduate & above	67	16.1
6.	Occupation of the mother	Government employee	6	1.4
		Private employee	16	3.8
		Self-employed	40	9.6
		Student	27	6.5
		Homemaker	327	78.7
		Retired	0	0
7.	Age of the father (years)	< 20	0	0
		21-30	182	43.8
		>30	234	56.3
8.	Education of the father	Illiterate	12	2.9
		Just literate	17	4.1
		Primary (5th)	25	6.0
		Junior high (8th)	68	16.3
		High school (10th)	96	23.1
		Intermediate (12th)	93	22.4
		Graduate & above	105	25.2
9.	Occupation of the father	Unemployed	58	13.9
		Government employee	44	10.6
		Non-government employee	67	16.1
		Self-employed	225	54.1
		Student	22	5.3
		Retired	0	0
10.	Religion	Hindu	358	86.1
		Muslim	58	13.9
		Others**	0	0
11.	Caste category	General	138	33.2
		OBC	208	50.0
		SC/ST/Others	70	16.8
12.	Family type	Nuclear	228	54.8
		Joint	154	37.0
		Three-generation	34	8.2
13.	Socioeconomic status (Modified BG Prasad) ***	Class I	0	0
		Class II	2	0.5
		Class III	164	39.4
		Class IV	172	41.3
		Class V	78	18.8

**Figure 1:**
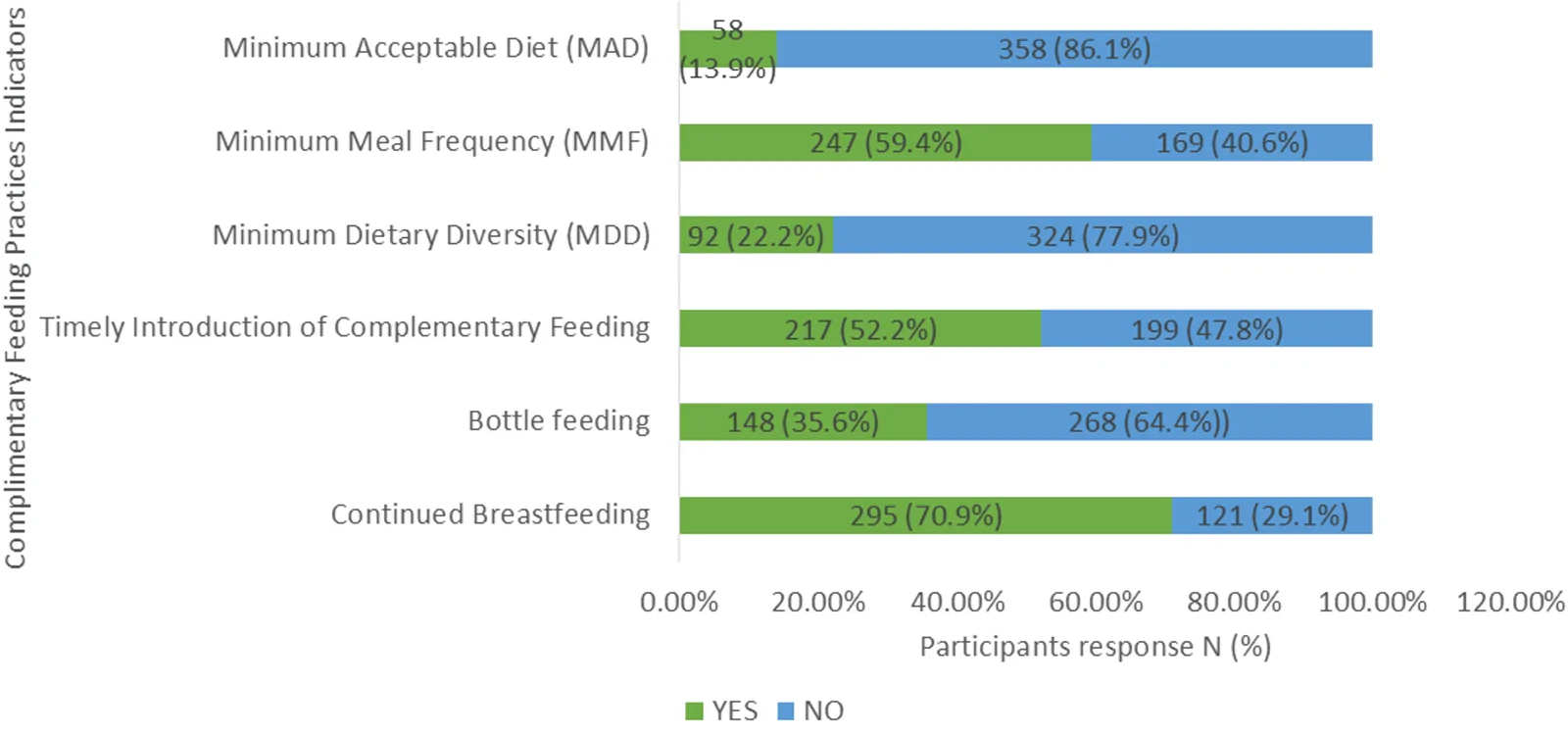
Distribution of complementary feeding practice indicators among study participants (N = 416)

Breastfeeding was still happening for 295 (70.9%) of children, but sub-optimal feeding practices were common, with children using bottle feeds (148, 35.6%) and complementary foods being introduced early or late in 199 (47.8%) of respondents. Dietary quality was also found to be very low, with 324 (77.9%) children not meeting the minimum dietary diversity recommendations, and only 169 (59.4%) meeting age-appropriate meal frequency recommendations. At the overall level, only 58 (13.9%) children were able to obtain the minimally acceptable diet, with 358 (86.1%) children in the study sample not achieving this basic dietary standard (Figure 1).

**Figure 2:**
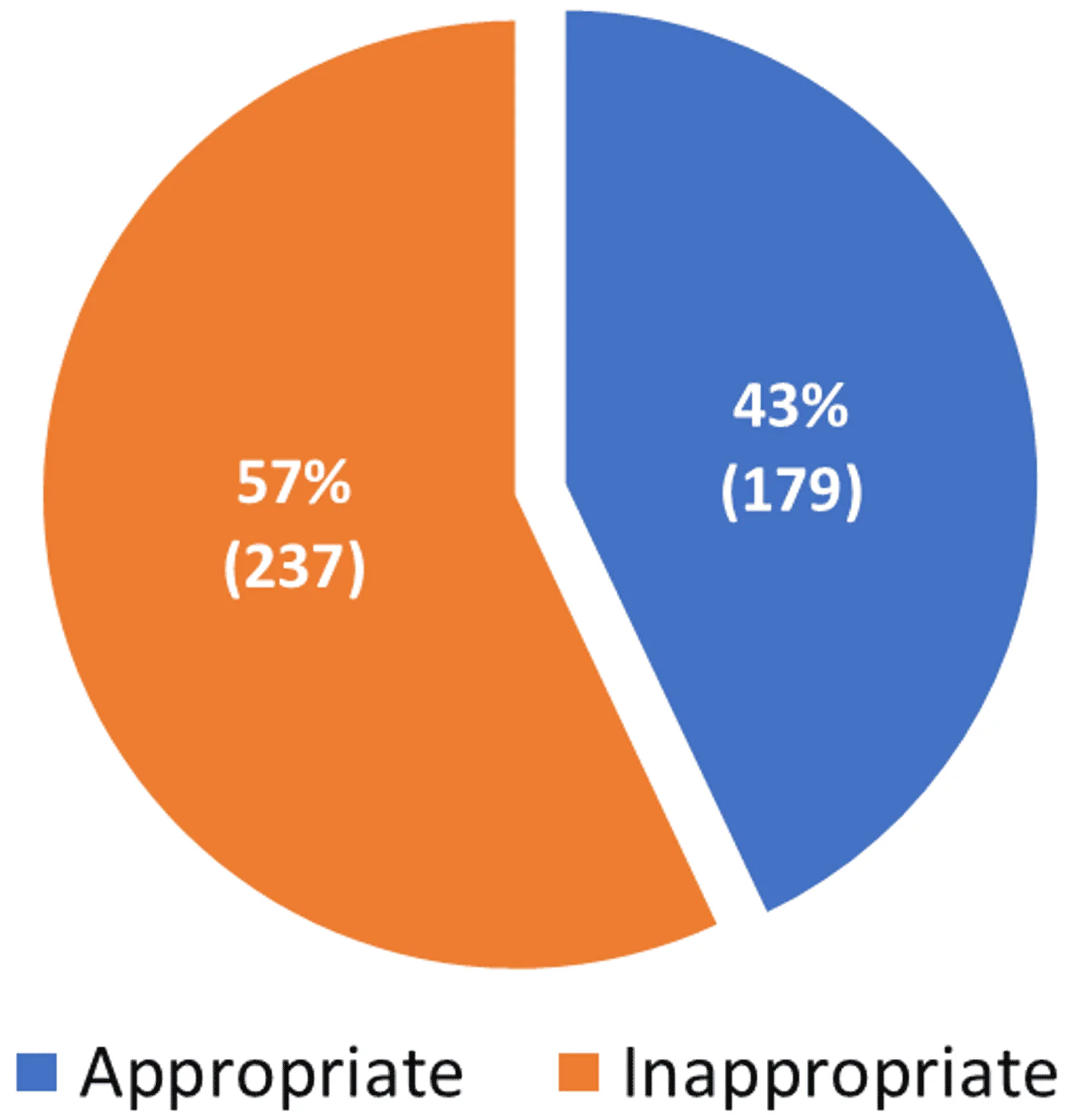
Prevalence of appropriate and inappropriate complementary feeding practices among study participants (N = 416)

CFI-based classification revealed appropriate complementary feeding in 179 (43.0%) children and inappropriate feeding in 237 (57.0%) children, confirming that the majority of young children in Etawah district are not receiving nutritionally adequate complementary care (Figure 2).

Table 2 summarises chi-square analyses examining the relationship between 13 sociodemographic variables and CFI-based feeding classification across the 416 participants, of whom 179 (43.0%) were categorised as receiving appropriate and 237 (57.0%) as receiving inappropriate complementary feeding. Statistical testing did not reveal a significant association between feeding adequacy and child sex (p = 0.537), birth order (p = 0.165), maternal age (p = 0.511), maternal education (p = 0.105), maternal occupation (p = 0.086), paternal age (p = 0.950), paternal educational level (p = 0.743), paternal occupation (p = 0.057), household religion (p = 0.559), or caste group (p = 0.844). In contrast, family type emerged as a statistically significant predictor (χ² = 6.595, p = 0.037): the proportion of children receiving appropriate feeding was the highest in nuclear households (111, 48.7%) and substantially lower in joint (56, 36.4%) and three-generational family settings (12, 35.3%). Socioeconomic status likewise showed a highly significant association (χ² = 11.615, p = 0.009), with appropriate feeding declining progressively from Class III (84, 51.2%) to Class V (22, 28.2%). Taken together, these results highlight household structure and economic class as the principal sociodemographic determinants of complementary feeding quality in this population.

**Table 2:** Association between sociodemographic factors and complementary feeding practices (N = 416) * NS = Not significant. ** χ² = Chi-square test (Fisher's exact test where expected count <5)

Variables	Category	Appropriate n (%)	Inappropriate n (%)	Total n (%)	χ² value	p-value	Significance
Gender	Male	95 (41.7)	133 (58.3)	228 (100)	0.382	0.537	NS
	Female	84 (44.7)	104 (55.3)	188 (100)			
Birth order	1	41 (36.6)	71 (63.4)	112 (100)	3.605	0.165	NS
	2	77 (48.1)	83 (51.9)	160 (100)			
	≥3	61 (42.4)	83 (57.6)	144 (100)			
Maternal age	<20 years	18 (36.7)	31 (63.3)	49 (100)	1.344	0.511	NS
	21-30 years	107 (45.1)	130 (54.9)	237 (100)			
	>30 years	54 (41.5)	76 (58.5)	130 (100)			
Maternal education	Illiterate	4 (23.5)	13 (76.5)	17 (100)	10.500	0.105	NS
	Just literate	3 (25.0)	9 (75.0)	12 (100)			
	Primary	16 (50.0)	16 (50.0)	32 (100)			
	Junior high	32 (36.4)	56 (63.6)	88 (100)			
	High school	38 (40.9)	55 (59.1)	93 (100)			
	Intermediate	56 (52.3)	51 (47.7)	107 (100)			
	Graduate+	30 (44.8)	37 (55.2)	67 (100)			
Maternal occupation	Govt. employee	1 (16.7)	5 (83.3)	6 (100)	8.158	0.086	NS
	Non-govt.	5 (31.3)	11 (68.8)	16 (100)			
	Self-employed	23 (57.5)	17 (42.5)	40 (100)			
	Student	15 (55.6)	12 (44.4)	27 (100)			
	Homemaker	135 (41.3)	192 (58.7)	327 (100)			
Paternal age	≤30 years	78 (42.9)	104 (57.1)	182 (100)	0.004	0.950	NS
	>30 years	101 (43.2)	133 (56.8)	234 (100)			
Paternal education	Illiterate	6 (50.0)	6 (50.0)	12 (100)	3.504	0.743	NS
	Just literate	7 (41.2)	10 (58.8)	17 (100)			
	Primary	9 (36.0)	16 (64.0)	25 (100)			
	Junior high	24 (35.3)	44 (64.7)	68 (100)			
	High school	41 (42.7)	55 (57.3)	96 (100)			
	Intermediate	42 (45.2)	51 (54.8)	93 (100)			
	Graduate+	50 (47.6)	55 (52.4)	105 (100)			
Paternal occupation	Unemployed	18 (31.0)	40 (69.0)	58 (100)	9.164	0.057	NS
	Govt. employee	22 (50.0)	22 (50.0)	44 (100)			
	Non-govt.	25 (37.3)	42 (62.7)	67 (100)			
	Self-employed	100 (44.4)	125 (55.6)	225 (100)			
	Student	14 (63.6)	8 (36.4)	22 (100)			
Religion	Hindu	152 (42.5)	206 (57.5)	358 (100)	0.341	0.559	NS
	Muslim	27 (46.6)	31 (53.4)	58 (100)			
Caste	General	60 (43.5)	78 (56.5)	138 (100)	0.340	0.844	NS
	OBC	87 (41.8)	121 (58.2)	208 (100)			
	SC/ST	32 (45.7)	38 (54.3)	70 (100)			
Family type	Nuclear	111 (48.7)	117 (51.3)	228 (100)	6.595	0.037	Significant
	Joint	56 (36.4)	98 (63.6)	154 (100)			
	Three-generation	12 (35.3)	22 (64.7)	34 (100)			
Socioeconomic status	Class II	1 (50.0)	1 (50.0)	2 (100)	11.615	0.009	Significant
	Class III	84 (51.2)	80 (48.8)	164 (100)			
	Class IV	72 (41.9)	100 (58.1)	172 (100)			
	Class V	22 (28.2)	56 (71.8)	78 (100)			

## Discussion

Data from this study confirm that supplementary feeding adequacy among children aged 6-23 months in Etawah district is substantially deficient, with the CFI recording appropriate practices in barely 43% of the cohort - a level consonant with the widespread suboptimality of child nutrition documented nationally and regionally across India. Continued breastfeeding was documented in 70.9% of children, exceeding the 67% reported by the National Family Health Survey 5 (NFHS-5) for the same age group in India [7], reflecting broadly supportive breastfeeding attitudes in this community; Sharma et al. reported similarly high breastfeeding rates in urban Uttar Pradesh, with 60% of children breastfed exclusively to six months and continuing thereafter [15]. A weaning rate of 29.1%, however, remains clinically concerning; WHO guidance recommends uninterrupted breastfeeding alongside complementary foods until at least two years of age, and early cessation is typically driven by perceived low milk supply, maternal employment demands, cultural practices, and weak household support systems [3]. Bottle feeding affected 35.6% of children - a proportion exceeding the 29% documented by Chhabra et al. in East Delhi [16] but well below the 60.7% reported in Saudi Arabia by Al-Mutairi et al. [17] - and carries recognised public health risks, including contamination-related enteric and respiratory infections, displacement of breast milk, and overconsumption-related obesity risk [18]. Bottle use is perpetuated by convenience-seeking, modernity perceptions, aggressive formula promotion, and limited caregiver knowledge of risks, highlighting the urgency of targeted counselling on cup and spoon feeding alternatives. Complementary foods were introduced at six months in 52.2% of children, surpassing the national NFHS-5 figure of 42.7% [7] yet leaving nearly half the sample subject to hazards of both premature introduction (infection risk, allergic sensitisation, reduced breastfeeding) and delayed introduction (growth faltering, micronutrient depletion); similar patterns were noted by Varghese et al. in peri-urban Lucknow [14], driven by entrenched cultural convictions, maternal knowledge gaps, and the normative authority of older family members especially in extended households. Dietary quality deficits were the most prominent finding: MDD was achieved by only 22.1% of children, and MAD by a mere 13.9%, reflecting heavy dependence on nutritionally incomplete cereal-based preparations, poor in protein, fresh produce, and animal-source ingredients. These proportions are broadly in line with the 11% MDD from Sankaran et al. in Uttar Pradesh [19], 19% MAD from Mulugeta et al. in Ethiopia [20], 11.3% MAD from NFHS-5 national data [7], and 9% MAD from Chhabra et al. [16]; somewhat higher rates of 17.1% were noted in Ghana by Ativor et al. [21], reflecting intersectoral economic and contextual variation. Drivers of poor dietary diversity are multi-layered - household poverty, limited maternal awareness of nutrition, food taboos around eggs and meat, seasonal supply constraints, and the prescriptive influence of elder household members. Kachwaha et al. calculated that a nutritionally adequate diet in Uttar Pradesh costs approximately US$904 annually, more than twice the cost of an energy-only diet (US$393), with around three-quarters of households unable to afford such expenditure [10], highlighting the formidable structural economic barrier. Age-appropriate meal frequency was achieved by 59.4% of children, while 40.6% received fewer meals than recommended; given the restricted gastric capacity and high energy needs of this age group, inadequate feeding frequency independently precipitates nutrient shortfalls and growth faltering regardless of food quality. Evidence from comparable resource-constrained settings corroborates these patterns: Ahmad et al. in Aceh, Indonesia, reported timely introduction in 50%, adequate meal frequency in 74%, dietary diversity in 50%, and MAD in 40% among 392 children [22]; Masuke et al. in northern Tanzania documented appropriate feeding in only 25.6% [23]; Twabi et al. in Malawi found that a mere 7.7% of children received appropriate feeding, with suboptimal practices associated with impaired growth and wasting [24]; and Na et al. using Pakistan DHS data observed 67% timely introduction but only 22% MDD and 15% MAD compliance [25]. Taken together, these cross-national comparisons underscore that deficient supplementary feeding is a pervasive challenge in lower-resource settings that demands context-specific, multi-pronged solutions grounded in local dietary habits, economic realities, and cultural frameworks.

There are many elements to the design that enhance the scientific rigor of the work. The community-based sampling framework with a balanced rural/urban representation offers better estimates of the population-level indicators relevant for district nutrition planning. There is a need for adequate statistical power, WHO-standardised anthropometry, as well as a validated instrument for comparison. The composite CFI allowed for multi-dimensional evaluation of feeding quality and layered quality assurance by daily checking of records, and 10% supervisory back-verification reduced measurement errors.

However, there are a number of caveats to make. The cross-sectional design does not allow causal relationships to be determined. The effective sample size did not consider the design effect in this study. The possibility of recall and social-desirability biases exists in 24-h maternal recall and seven-day food-frequency data, when assessing diet history, even consent/volunteer bias, as only those who had provided consent were included. The biochemical micronutrient status was not measured and included; seasonality of food supplies and consumption were not captured, and results may require reinterpretation for use with other populations outside of Etawah.

## Conclusions

The study clearly demonstrates that the feeding of supplementary foods to children aged 6-23 months is way below optimum in Etawah district, where only 43% of children are being fed according to the CFI. The largest deficiencies are in dietary diversity (MDD: 22.1%) and MAD (13.9%), which are both indicative of high levels of reliance on nutritionally poor, cereal-based diets. Household structure and household economic class were the only sociodemographic correlates found to be significant for feeding quality. It is important to address these gaps to not just support child health but also to contribute to the national nutrition agenda in India and meet SDG-2 (Zero Hunger) and SDG-3 (Good Health and Well-Being). To move towards meaningful and sustained improvement in feeding practices, a systemic response is needed, including caregiver nutrition literacy, upskilling frontline health workers, socioeconomic enablement strategies, and robust monitoring systems.

The study results suggest the following recommendations for the program. First, demand-side nutrition literacy should be increased via regular education events at the village level, on dietary diversity, meal sufficiency, and how to make cost-effective nutrient-rich foods from local produce; reach can be extended using broadcast and digital media, and illustrated counselling tools can be developed for use by front-line workers. Second, capacity investment in the service providers is required through structured induction and refresher training of Anganwadi workers, accredited social health activists, and auxiliary nurse midwives, through supportive supervision and standardised home-visit assessment procedures. Third, the gaps in access to food should be closed through increased food supplementation under ICDS, household vegetable gardens, backyard poultry schemes, and the establishment of community-based therapeutic nutrition centres. Fourth, there is a need to risk stratify resources allocation, with preference to the least educated parents, households with extended families, high bottle-feeding index, and early termination of breastfeeding. Fifth, tracking mechanisms need to include periodic nutrition data collection via nutrition surveys at the population level, in combination with routine CFI-based performance indicators on district health information platforms. Sixth, a policy intervention is needed that connects nutrition programmes with income generation programmes, social safety nets, and policies and programmes that allow for maternal labour involvement and breastfeeding support at the workplace. Seventh, the role of community members should be mobilised using self-help groups, representatives at the village level, and village leaders, and community engagement should be promoted as a means of tackling cultural barriers through participatory approaches. Finally, funding efforts to carry out applied research, the local production of fortified foods at affordable prices, and mobile-health platforms for interactive nutrition counselling will maintain the behavioural change over time.

## Appendices

**Table 3:** Sociodemographic profile of the participants

S. No.	PARTICULARS	RESPONSE/CODE
A.1	Residential area: 1. Rural, 2. Urban	
A.2	Name of village/ward	
A.3	Name of ASHA and phone number	
A.4	Name of child	
A.5	Mother’s name	
A.6	Father’s name	
A.7	Phone number of either parent	
Profile of Child		
A.8	Age of child (month): ___	
A.9	Gender of child: 1. Male, 2. Female	
A.10	Birth order of the child:	
Profile of Mother		
A.11	Age of mother (years):	
A.12	Education of the mother: 1. Illiterate, 2. Just literate, 3. Primary school (5th), 5. Junior high school (8th), 6. High school (10th), 7. Intermediate (12th), 8. Graduate & postgraduate	
A.13	Occupation of the mother: 1. Unemployed, 2. Government Employee, 3. Non-government Employee, 4. Self-Employed, 5. Student, 6. Homemaker, 7. Retired/Pensioner	
Profile of Father		
A.14	Father’s age (years)	
A.15	Education of the father: 1. Illiterate, 2. Just literate, 3. Primary school (5th), 5. Junior high school (8th), 6. High school (10th), 7. Intermediate (12th), 8. Graduate & postgraduate	
A.16	Occupation of the father: 1. Unemployed, 2. Government Employee, 3. Non-government Employee, 4. Self-Employed, 5. Student, 6. Retired/Pensioner	
Family Profile		
A.17	Religion: 1. Hindu, 2. Muslim, 3. Others: Christian, Jain, Buddhist, Parsi, etc.	
A.18	Caste: 1. General, 2. OBC, 3. SC/ST/Others	
A.19	Number of family members:	
A.20	Family type: 1. Nuclear, 2. Joint, 3. Three generation	
A.21	Monthly income of the family from all sources (in rupees)	
A.22	Per capita monthly income (in rupees)	
A.23	Socioeconomic class as per Modified B G Prasad Classification: 1. Class I (Upper Class), 2. Class II (Upper Middle Class), 3. Class III (Middle Class), 4. Class IV (Lower Middle Class), 5. Class V (Lower Class)	

**Table 4:** Complimentary feeding index *Scoring of CFI was done by assigning (i) Score 2 for good practice and (ii) Score 1 for bad practice. *The mean CFI score was calculated, and then the individual score was compared with the mean score. If the individual score ≥ the mean score = appropriate complementary feeding; if the individual score < the mean score = inappropriate complementary feeding. Note: The tool was self-generated based on the WHO guideline for complementary feeding of infants and young children 6-23 months of age [26].

S. No.	VARIABLES	RESPONSES		CODE
B.1	Do you still breastfeed your child?	Yes-2		
		No-1		
B.2	Do you use a bottle to feed your child?	Yes-1		
		No-2		
B.3	Had you started complementary feeding at 6 months of age for your child?	Timely-2		
		Not timely-1		
B.4	Dietary Diversity Score (24-Hour Recall): Among the following food groups, which foods did you feed your child in the past 24 hours? 1. Starchy staples: breads (e.g., chapatti, rolls) ( ), biscuit ( ), porridge ( ), noodles (e.g., pasta, vermicelli) ( ), rice ( ), rye ( ), barley ( ), corn (maize) ( ), millet ( ), oats ( ), potato ( ), others ( ); 2. Pulses, legumes, and nuts: arhar ( ), channa ( ), matar ( ), peanuts ( ), beans ( ), lentils ( ), soybeans ( ), others ( ); 3. Milk (other than breast milk) and milk products: milk ( ), cheese ( ), yogurt ( ), others ( ); 4. Flesh foods: chicken ( ), goat ( ), beef ( ), pork ( ), others ( ); 5. Vitamin A-rich fruits and vegetables: ripe mango ( ), ripe papaya ( ), carrot greens ( ), amaranth greens ( ), bitter gourd ( ), beet greens ( ), broccoli ( ), others ( ); 6. Eggs ( ); 7. Others ( )	6-8 months	0-2 food group -1	
			≥3 food groups -2	
		9-23 months	0-3 food group -1	
			≥4 food groups -2	
			B.5. Food group frequency score (past 7-day food frequency questionnaire)				
B.5.a	How many times in the past week did you include starchy staples (grain/tubers: e.g., breads (e.g., chapatti, rolls), biscuit, porridge, noodles (e.g., pasta, vermicelli), rice, rye, barley, corn (maize), millet, oats, potato, others)	6-8 months	0-3 times -1	
			≥4 times - 2	
		9-23 months	0-4 times -1	
			≥5 times -2	
B.5.b	How many times in the past week did you include pulses, legumes, or nuts in a meal for your child? (arhar, channa, matar, peanuts, beans, lentils, soyabeans, others)	6-8 months	0-3 times -1	
			≥ 4 times -2	
		9-23 months	0-4 times -1	
			≥ 5 times -2	
B.5.c	How many times in the past week did you include milk or milk products in a meal for your child? (cheese, yogurt, others)	6-8 months	0-3 times -1	
			≥4 times - 2	
		9-23 months	0-4 times -1	
			≥ 5times - 2	
B.5.d	How many times in the past week did you include flesh food (meat, egg) in a meal for your child? (any meat, such as beef, pork, goat, chicken, others)	6-8 months	0-3 times -1	
			≥ 4times - 2	
		9-23 months	0-4 times -1	
			≥5 times -2	
B.5.e	How many times in the past week did you include vitamin A-rich fruits and vegetables in your child's meals? Ripe mango, ripe papaya, carrot greens, amaranth greens, bitter gourd, beet greens, broccoli, and others.	6-8 months	0-3 times -1	
			≥4 times - 2	
		9-23 months	0-4 times -1	
			≥5times -2	
B.5.f	How many times in past week, did you include “Other” fruits and vegetables in meal of your child?	6-8 months	0-3 times -1	
			≥4times - 2	
		9-23 months	0-4 times -1	
			≥5times -2	
B.5.g	How many times in the past week did you feed your child foods made with oil, fat, or butter in your child's meals?	6-8 months	0-3 times -1	
			≥4times - 2	
		9-23 months	0-4 times -1	
			≥5 times -2	
B.6	Meal frequency (past 24 h): In the past 24 hours, how many times did you feed your child?	6-8 months	0-2 times-1	
			≥3 times-2	
		9-23 months	0-3 times-1	
			≥4 times-2	
